# Is early thrombectomy of proximal middle cerebral artery occlusion to salvage internal capsule associated with improved clinical outcomes?

**DOI:** 10.3389/fstro.2026.1751007

**Published:** 2026-02-12

**Authors:** Elochukwu Ibekwe, Robert Kassinger, Nicholas Mannix, Jing Peng, Archana Hinduja

**Affiliations:** 1 Department of Neurology, The Ohio State University Wexner Medical Center, Columbus, OH, United States; 2 Department of Biostatistics, The Ohio State University Wexner Medical Center, Columbus, OH, United States

**Keywords:** internal capsule, lenticulostriate artery, middle cerebral artery, stroke, thrombectomy

## Abstract

**Background:**

Eloquence of tissue rather than infarct volume is a better predictor of outcomes following proximal middle cerebral artery (MCA) occlusion. The aim of this study was to determine the impact of white matter tract involvement, specifically the internal capsule (IC), following occlusion of non-collateralized lenticulostriate arteries (LSAs) on functional outcomes.

**Methods:**

A retrospective observational single-center study of patients with proximal MCA occlusions in the period from 2015 to 2020 who were treated with mechanical thrombectomy and had post-interventional diffusion-weighted imaging was conducted. Patients were distributed based on the presence or absence of IC infarction (IC+ vs. IC−) at the level supplied by the MCA LSAs. Multivariate logistic or linear regression analysis was used to evaluate factors associated with the development of IC infarction.

**Results:**

Of 368 patients with proximal MCA occlusion, 200 (55%) developed IC+ infarction. On univariate analysis, patients with IC+ infarction had higher baseline NIHSS (National Institute of Health Stroke Scale), lower ASPECTS (Alberta Stroke Program Early CT Score), lower collateral score, and were less likely to have partial reperfusion of LSA prior to thrombectomy. On multivariate analysis, those with higher baseline NIHSS, low ASPECTS, lack of successful reperfusion (TICI2b, 2c, 3), poor collateral circulation, and/or lack of partial perfusion of LSA prior to thrombectomy were likely to develop IC+ infarction. After adjusting for confounders, patients with IC+ infarction were less likely to experience early neurological improvement, more likely to develop hemorrhagic transformation of putamen, and had larger infarct volume. However, no significant correlation between IC+ infarction with poor 3-month functional outcome was observed.

**Conclusion:**

Delayed recovery is possible despite IC+ infarction and hemorrhagic transformation of lenticulostriate territory. Larger studies are needed to confirm these findings.

## Introduction

Ischemic stroke secondary to proximal middle cerebral artery (MCA) occlusions is the most common type of stroke, accounting for 50% of intracranial occlusions (Hansen et al., 2015). Prior to utilization of mechanical thrombectomy, this clinical syndrome was associated with poor clinical outcomes (Hacke et al., 1996; Heinsius et al., 1998). With current advances in endovascular mechanical thrombectomy techniques and tools, outcomes following large vessel occlusions have improved (Garcia-Esperon et al., 2022). Nevertheless, several factors such as location of arterial vascular occlusion, eloquence of the infarcted tissue, degree of collateral flow, time to recanalization, degree of revascularization, number of thrombectomy passes, initial National Institutes of Health Stroke Scale (NIHSS), baseline functional status, and systemic comorbidities have influence on the functional outcome following endovascular thrombectomy (Behme et al., 2014; Liggins et al., 2015; Ringheanu et al., 2023). Of these factors, location of occlusion and eloquence of infarcted tissue (particularly cerebral white matter tract) are important factors to consider. When compared to gray matter, the white matter is resistant to hypoxic ischemic injury, resulting in variability in stroke severity and clinical outcomes compared to gray matter. Therefore, timely endovascular thrombectomy of MCA occlusions could salvage the white matter tracts and potentially improve functional outcomes (Falcao et al., 2004; Bristow et al., 2005; Koga et al., 2005; Nagakane et al., 2008). Furthermore, proximal MCA occlusion is associated with worse clinical outcomes compared to distal M1 occlusion because of infarction of the striatocapsular tissue from obstruction of the non-collateralized lenticulostriate arteries (LSA; Loh et al., 2009; Behme et al., 2015; Kaesmacher et al., 2021). However, other studies reported that infarction of the lenticulostriate territories from proximal M1 occlusion was not associated with poor functional outcomes (Ko, 2021).

We hypothesize that early endovascular thrombectomy of proximal M1 occlusion could reperfuse the LSA and salvage the pyramidal white matter tract that could lead to improved functional outcomes. In this retrospective study, we attempted to explore the risk factors and the impact of internal capsule (IC) infarction on 3-month functional outcome following endovascular thrombectomy for proximal MCA occlusion in our patient population.

## Methods

### Study population

A retrospective review from our prospectively collected database of all consecutive patients who underwent endovascular thrombectomy from January 2015 to December 2020 at a tertiary care academic center was performed. All patients aged 18 years and above with isolated MCA occlusions that underwent a high-resolution diffusion-weighted imaging/apparent diffusion correlate (DWI/ADC) magnetic resonance imaging (MRI) sequences following endovascular thrombectomy were included. This study was approved by our local institution review board.

### Parameters of assessment

The demographic data included patient demographics, medical comorbidities, clinical status, thrombectomy parameters, radiological findings, and functional outcome. Functional outcome was determined using 3-month mRS (modified Rankin scale), with mRS <2 indicating a good functional outcome. The ASPECTS (Alberta Stroke Program Early CT Score) and collateral score were calculated using computed tomography (CT) head and CT angiogram (CTA) on admission, respectively. Collateral score was calculated using the grading score proposed by Tan et al. (2009), which ranges from 0 to 3, with grade 0 for 0% filling of the occluded vascular territory, grade 1 for >0% but < 50% filling of occluded vascular territory, grade 2 for > 50% but < 100% filling of occluded vascular territory, and grade 3 for 100% filling of the occluded vascular territory.

Assessment of proximal vs. distal MCA occlusion was assessed using interventional diagnostic cerebral angiogram (DCA) prior to thrombectomy. Proximal MCA occlusion was defined as occlusion of the LSA, while distal MCA occlusion spared the LSA. Infarction and hemorrhage of striatocapsular regions was assessed using follow-up MRI after endovascular intervention.

### Image analysis

**Follow-up MRI**. Each brain MRI was reviewed for the presence of IC infarction, specifically where the pyramidal fibers descend shortly before and traversing the caudate and putamen. This level of IC infarction corresponds to the area supplied by the posterio-superior LSA (Djulejic et al., 2016). The MRI diffusion-weighted image with apparent diffusion correlate (DWI/ADC) sequences were used to assess for the presence (IC+) or absence (IC-) of IC infarction. Susceptibility weighted imaging sequence was used to assess for hemorrhage in the putamen. Infarct volume was calculated using the sum of manually delineated borders of individual infarcts slice multiplied by the slice thickness (Oppenheim et al., 2000; Aoki et al., 2015). All imaging parameters were reviewed by two neurologists and in the case of discrepancy, a consensus read was done by a neuroradiologist.

**Figure 1:**
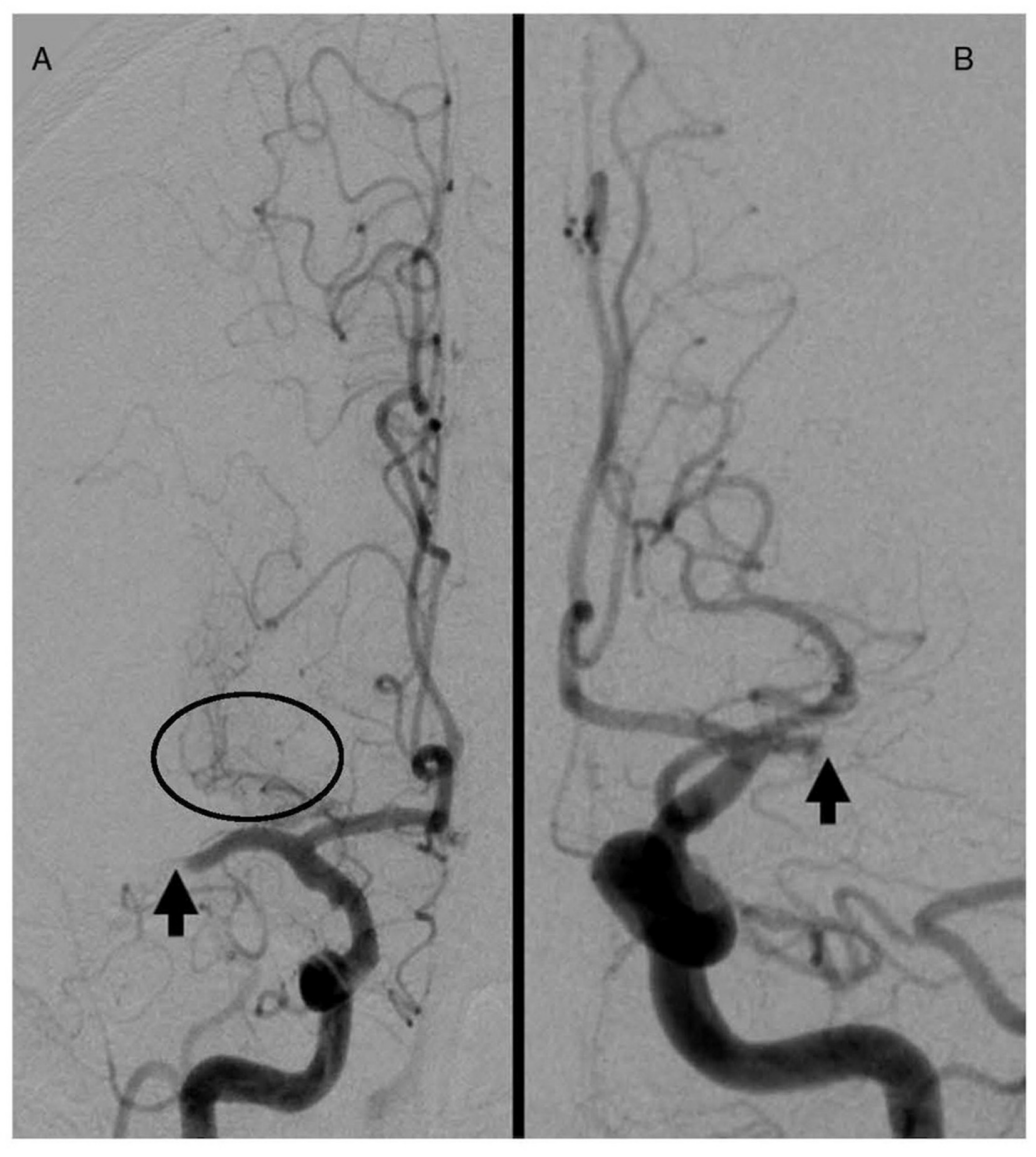
**(A)** Distal MCA occlusion sparing the LSA (circle). **(B)** Proximal MCA occlusion occluding LSA. Arrows indicate site of occlusion.

**Digital subtraction angiography**. Patency of LSA was evaluated using the first run images obtained during DCA prior to thrombectomy. Lack of visualization or visualization of only the most proximal LSA was indicative of complete occlusion, and patency of medial or lateral LSA was indicative of partial perfusion or lack of occlusion (Kaesmacher et al., 2021; Figure 1). The thrombolysis in cerebral infarction score (TICI) was used to assess recanalization status of the occluded MCA. A consensus read was done by a neuroradiologist for any discrepancy. Successful reperfusion was defined as a TICI score of 2b, 2c, or 3.

### Clinical outcome

Functional outcome was assessed using mRS at discharge and at 3 months. Functional independence at discharge was defined as mRS ≤ 2 at discharge. Good functional outcome was defined as mRS ≤ 2 at 3 months. Early neurological improvement was defined as either an NIHSS of ≤ 1 or an improvement by ≥8 upon discharge when compared to the initial score.

### Statistical analysis

Baseline characteristics of demographic variables and clinical features were compared between patients with and without IC strokes. For normally distributed continuous variables, the mean with standard deviation was used with a two-sample *t*-test for comparing the two groups. For non-normally distributed continuous variables, the median with interquartile range (IQR) was used with the Wilcoxon rank sum test. For categorical variables, count and frequency for each level were shown and compared using chi-square test if the sample size is large, while Fisher's exact test was used with small sample size. Important prognostic variables were selected for fitting a multivariate logistic regression model and further filtered using stepwise selection. We also performed logistic regression analysis between IC stroke status and categorical clinical outcomes, and linear regression analysis between IC stroke status and continuous clinical outcomes. A *p*-value of < 0.05 was considered to be significant. All unadjusted group comparisons were performed for exploratory purposes; therefore, *p*-values were not adjusted for multiple comparisons. Variables significant on univariate analysis (*p* < 0.05) and clinically relevant variables were entered in the multivariate analysis. All statistical analysis were performed using software package R (version 4.2.0, R Core Team, 2022).

## Results

Of 368 patients (mean age 65 years, 54.1% women) with proximal MCA occlusions that met our inclusion criteria, 200 patients (54.3%) had IC+ infarction. Successful reperfusion of LSA was achieved in 356 patients (95.0% of IC+, and 98.8% of IC- infarctions). The demographics and clinical features are described in Table 1. The baseline median NIHSS for patients with IC+ and IC- infarction was 16 (IQR 11–21) and 12 (IQR 7–18) with improvement to 11 and 5 (IQR 4–17, and 1–11) at day 7 or at discharge, respectively. Patients with IC+ infarction had significantly higher NIHSS, lower ASPECT score, lower CTA collateral score, and less likely had partial perfusion of LSA prior to thrombectomy (Table 1). Of the 12 patients without successful reperfusion of LSA, 10 developed IC+ infarction.

**Table 1:** Baseline characteristics with strata of internal capsule ischemia.

**Variables**	**Sparing IC (IC–) (168 patients)**	**Involving IC (IC+) (200 patients)**	***P*-value**
Age, years, mean (SD)	65.95 (14.70)	66.17 (14.69)	0.888
Sex, female (%)	84 (50.0)	115 (57.5)	0.183
Diabetes mellitus (%)	49 (29.2)	57 (28.5)	0.980
Hypertension (%)	122 (72.6)	149 (74.5)	0.772
Atrial fibrillation (%)	50 (29.8)	67 (33.5)	0.513
History of stroke/TIA (%)	28 (16.5)	31 (15.5)	0.872
IV rtPA (%)	80 (47.6)	79 (39.7)	0.156
Baseline NIHSS (median [IQR])	12.00 [7.00, 18.00]	16.00 [11.00, 21.00]	< 0.001
ASPECTS (median [IQR])	9.00 [8.00, 10.00]	8.00 [6.00, 9.00]	< 0.001
CTA-collaterals (median [IQR])	2.00 [1.00, 2.00]	2.00 [1.00, 2.00]	0.009
Partial perfusion of LSA on admission (%)	147 (87.5)	118 (59.0)	< 0.001
Successful reperfusion of LSA after MT (%)	166 (98.8)	190 (95.0)	0.073
Symptom onset to groin puncture, minutes (median [IQR])	312.00 [207.50, 556.50]	310.00 [213.00, 752.00]	0.265
Symptom onset to final reperfusion, minutes (median [IQR])	335.00 [246.50, 630.50]	342.50 [258.00, 771.00]	0.242
ICAD (%)	33 (19.6)	29 (14.5)	0.241
**Successful reperfusion (%)**			0.086
TICI 2b, 2c	83 (54.2)	108 (64.3)	
TICI 3	70 (45.8)	60 (35.7)	
Time to follow-up MRI, days (median [IQR])	1.00 [1.00, 2.00]	1.00 [1.00, 2.00]	0.058
Baseline mRS	0.57 (0.97)	0.50 (0.86)	0.494
**TOAST**			0.694
Cardioembolic	79 (47.0)	110 (50.0)	
Cryptogenic	59 (35.1)	61 (30.5)	
LVD	19 (11.3)	28 (14.0)	
Other known causes	11 (6.5)	11 (5.5)	
**Onset to recanalization**			1.000
< 6 h	91 (55.8)	105 (55.3)	
>6 h	72 (44.2)	85 (44.7)	

IC, internal capsule; TIA, transient ischemic attack; NIHSS, National Institute of Health stroke scale; ASPECTS, Alberta stroke program early CT score; IV rtPA, intravenous recombinant tissue plasminogen activator; CTA, computed tomography angiography; LSA, lenticulostriate artery; MT, mechanical thrombectomy; IQR, interquartile range; TICI, thrombolysis in cerebral infarction; MRI, magnetic resonance imaging; LVD, large vessel disease; ICAD, intracranial atherosclerotic disease.

We included 10 covariates: age, sex, baseline NIHSS, ASPECT score, CTA-collaterals, partial perfusion of LSA on admission, intracranial atherosclerotic disease, symptom onset to recanalization, time to follow-up imaging, and successful reperfusion (TICI 2b, 2c, 3) in the multivariate analysis (Table 2). In our study, after adjusting for other covariates, males showed a non-significant trend toward a lower odds of developing IC+ infarction (adjusted OR 0.59; 95% CI: 0.33–1.04; *p* = 0.067). For the baseline NIHSS score, one unit increase led to a 7% increase in the odds of IC+ infarction (adjusted OR 1.07; 95% CI: 1.03–1.12; *p* = 0.001). Similarly, a lower ASPECT score increased the odds of IC+ infarction (adjusted OR 0.6; 95% CI: 0.49–0.74; *p* < 0.001). Patients with partial perfusion of LSA on admission had lower odds of IC+ compared to those without partial perfusion on admission (adjusted OR 0.14; 95% CI: 0.07–0.28; *p* < 0.001). Patients with successful reperfusion were less likely to develop IC+ infarction (adjusted OR 0.54; 95% CI: 0.31–0.94; *p* = 0.029). A borderline significance between CTA collateral score with development of IC+ infarction was observed (adjusted OR 0.6; 95% CI: 0.36–0.99; *p* = 0.046).

**Table 2:** Multivariate regression analysis of factors associated with internal capsule infarction.

**Variables**	**Odds ratio**	**95% CI**	***P*-value**
Age	1	1–1	0.334
Sex (male vs. female)	0.59	0.33–1.04	0.067
Baseline NIHSS	1.07	1.03–1.12	<0.001
ASPECTS	0.6	0.49–0.74	<0.001
CTA-collaterals	0.6	0.36–0.99	0.046
Partial perfusion of LSA on admission	0.14	0.07–0.28	<0.001
Successful reperfusion (TICI2b, 2c, 3)	0.54	0.31–0.94	0.029
Time to follow-up MRI (days)	1.16	0.93–1.44	0.188
Symptom onset to recanalization	0.99	0.98–1.01	0.534
ICAD	0.53	0.25–1.1	0.087

NIHSS, National Institute of Health stroke scale; ASPECTS, Alberta stroke program early CT score; CTA, computed tomography angiography; LSA, lenticulostriate artery; TICI, thrombolysis in cerebral infarction; CI, confidence interval; ICAD, intracranial atherosclerotic disease.

When compared to the IC- group, patients in IC+ were more likely to experience hemorrhagic transformation of the putamen (45.3% vs. 12.0%, *p* < 0.001), had a lower likelihood of early neurological improvement (43.5% vs. 63.1%, *p* < 0.001), were less likely to achieve functional independence at discharge (16.5% vs. 30.4%, *p* < 0.002), had larger infarct volume (61.9 cc [IQR, 19.14–166.55] vs. 19.87 cc [4.74–64.10], *p* < 0.001), higher NIHSS at 7 days or discharge (11 [IQR, 4–17] vs. 5 [IQR, 1–11], *p* < 0.001), higher mRS at discharge (4 [IQR, 3–4] vs. 3 [IQR, 2–4], *p* < 0.001), and were less likely to achieve good functional outcome at 3 months (28.3% vs. 42.9%, *p* = 0.011; Table 3). After adjusting for age, symptom onset to recanalization, intracranial atherosclerosis, baseline NIHSS, ASPECT score, CTA-collaterals, and partial perfusion of LSA on admission on multivariate regression analysis, IC+ infarction was associated with increased odds of developing hemorrhagic transformation (adjusted OR 4.75; 95% CI: 2.58–8.75; *p* < 0.001), and lower likelihood of early neurological improvement (adjusted OR 0.48; 95% CI: 0.29–0.81; *p* = 0.005; Table 4). Patients with IC+ infarctions had larger infarct volumes (adjusted mean difference 37.57; *p* = 0.004), higher NIHSS score on day 7 or discharge (adjusted mean difference mean difference 2.66; *p* = 0.002), and higher mRS at discharge (adjusted mean difference 0.49; *p* = 0.003). However, no significant difference in functional status at discharge (OR 0.58; 95% CI: 0.32–1.07; *p* = 0.083), good functional outcomes at 3 months (OR 0.58; 95% CI: 0.32–1.07) and mRS at 3 months (OR 0.46; 95% CI: −0.03–0.95; *p* = 0.068) following IC+ infarction were observed.

**Table 3:** Association between internal capsule infarction and outcomes.

**Variables**	**Sparing IC (IC–)**	**Involving IC (IC+)**	***P*-value**
Hemorrhagic transformation of putamen (%)	20 (12.0)	87 (45.3)	<0.001
Early neurological improvement (%)	106 (63.1)	87 (43.5)	<0.001
Functional independence at discharge (mRS ≤ 2) (%)	51 (30.4)	33 (16.5)	<0.002
Good outcome at 3 months (mRS ≤ 2) (%)	60 (42.9)	47 (28.3)	0.011
Infarct volume, cc (median [IQR])	19.87 [4.74, 64.10]	61.90 [19.14, 166.55]	<0.001
NIHSS on day 7 or discharge (median [IQR])	5.00 [1.00, 11.00]	11.00 [4.00, 17.00]	<0.001
Discharge mRS, median [IQR]	3.00 [2.00, 4.00]	4.00 [3.00, 4.00]	<0.001

NIHSS, National Institute of Health stroke scale; IQR, interquartile range; mRS, modified Rankin scale; IC, internal capsule.

**Table 4:** Regression of internal capsule infarction and outcomes.

**Variables**	**Estimate**	**95% CI**	***P*-value**
Hemorrhagic transformation of Putamen (%)	4.75 (odds ratio)	2.58–8.75	<0.001
Early neurological improvement (%)	0.48 (odds ratio)	0.29–0.81	0.005
Functional independence at discharge (mRS ≤ 2)	0.58 (odds ratio)	0.32–1.07	0.083
Good outcome at 3 months (mRS ≤ 2)	0.58 (odds ratio)	0.32–1.07	0.082
Infarct volume, cc (median [IQR])	37.57 (mean difference)	12.09–63.05	0.004
NIHSS on day 7 or discharge (median [IQR])	2.66 (mean difference)	0.97–4.35	0.002
Discharge mRS, median [IQR]	0.49 (mean difference)	0.17–0.81	0.003
mRS at 3 months	0.46 (mean difference)	(-0.03–0.95)	0.068

NIHSS, National Institute of Health stroke scale; IQR, interquartile range; mRS, modified Rankin scale.

## Discussion

In our study, although patients with IC+ infarctions had poor mRS at discharge, this did not translate to worse long-term functional outcome. This is consistent with prior reports that demonstrated good outcome following thrombectomy despite proximal M1 occlusion (Kim et al., 2021; Ko, 2021). On the contrary, other studies demonstrated that sparing of LSA protects the IC and is associated with good functional outcomes (Behme et al., 2015; Liu et al., 2020; Kaesmacher et al., 2021). In our study, the lack of correlation between site of occlusion and functional outcome may be from initial thrombus obstruction of the proximal MCA perforators, followed by spontaneous distal migration resulting in distal M1 occlusion (Kim et al., 2021). Studies have reported that baseline ischemic core is a more powerful predictor of functional outcome than the site of occlusion itself (Tian et al., 2019). Clinical recovery of motor and sensory functions depends on the extent of injury to areas adjacent to the IC such as the basal ganglia, corona radiata, which influences overall outcome (Lee et al., 2017; Sato et al., 2020). The basal ganglia, which is also supplied by the LSA, is vulnerable to infarction and hemorrhagic transformation and has a variable outcome despite recanalization (Loh et al., 2009; Horie et al., 2020). Patients with isolated striatocapsular infarcts have favorable outcomes compared than those with non-isolated striatocapsular infarcts following thrombectomy of proximal MCA occlusions (Kaesmacher et al., 2017a). In addition to lesion location, hemispherical differences, unequal efficacy of different infarct locations, and the extent of neuronal injury also influence functional outcomes; however, these factors were not explored in our study (Fries et al., 1993; Rosso et al., 2019; Panni et al., 2020; Okamoto et al., 2021; Li et al., 2022). Diffusion tensor imaging studies assessing corticospinal tract integrity predicted motor recovery more accurately than clinical scores (Puig et al., 2011). Voxel-based studies showed that the left hemispherical lesions in the deep periventricular white matter and adjacent IC have a great impact on functional outcome (Ernst et al., 2018). We also did not analyze the extent of infarcted regions beyond the IC that may have impacted clinical outcomes.

The blood supply to the striatocapsular region is heterogeneous. The non-collateralized lateral LSA arises from the lateral MCA and solely supplies the dorsal IC genu and posterior limb of IC (at the level of caudate body and putamen; Feekes and Cassell, 2006; Decavel et al., 2012; Djulejic et al., 2016). The lower genu, and the inferior half of the posterior limb of IC at the level of the thalamus is supplied by a mixture of recurrent artery of Heubner, posterior communicating artery perforators, and anterior choroidal artery (Kaesmacher et al., 2021). Thus, an occlusion of the proximal MCA that involves the LSA is unlikely to incapacitate the entire IC, but it causes significant damage to the superior aspect of posterior limb of IC. Salvaging the posterior dorsal aspect of IC is time dependent, and the likelihood of infarction following MCA occlusion is high after 5 h (Kaesmacher et al., 2021). However, this association was not observed in our study, likely because we are a tertiary referral center which resulted in prolonged time from symptom onset to groin puncture exceeding 5 h in both groups that contributed to the lack of correlation. These findings highlight the importance of rapid, high-quality reperfusion of the MCA to prevent infarction of the dorsal and posterior aspect of the IC at the level of the caudate and putamen. Despite partial perfusion of LSA on admission, 118 patients developed IC+ infarction possibly from thrombus migration, with prolonged time to recanalization compared to other studies (Kaesmacher et al., 2017b, 2021). Our study demonstrated a reduced likelihood of IC+ infarction among patients with successful reperfusion and a favorable trend with good collateral circulation. This borderline trend may be attributed to the non-collateralized vascular supply of the superior aspect of posterior limb IC by the LSA (Kaesmacher et al., 2021). Similar to other studies, IC+ infarction was associated with high admission NIHSS (Kaesmacher et al., 2021). Our study identified a significant association between absence of partial reperfusion of LSA on admission and lower ASPECT score with the development of IC+ infarction, a relationship that was not explored in previous studies. It is possible that inadequate LSA perfusion prior to thrombectomy resulted in infarction of the deep white matter, including the IC, thereby contributed to a lower ASPECT score. In patients with large core infarctions, IC involvement accounted for more than 50% of impact of ASPECTS on functional outcome (Lee et al., 2026). Conversely, patients who demonstrated reversal of IC lesions had lower likelihood of poor outcomes. Variability across studies may reflect differences in patient populations and sample sizes, and these findings warrant confirmation in larger cohorts.

Unlike other studies, IC+ infarction was associated with large infarct volumes (Kaesmacher et al., 2021). Larger infarct volumes in our study may be attributed to the lack of partial LSA perfusion prior to thrombectomy, insufficient collateral circulation, and failure to achieve successful reperfusion. While the final infarct volume impacts the functional outcome, it only partially mediates the relationship between mechanical thrombectomy and 3-month functional outcome (Boers et al., 2019). Involvement of eloquent areas such as IC are associated with higher NIHSS and poor 3-month functional outcome (Habegger et al., 2018). In our study, although patients with IC+ infarction had greater infarct volume and hemorrhagic transformation this did not translate to poor 3-month functional outcome. This may be from neuronal plasticity or the fact that only 12% of treatment benefit comes from a reduced infarct volume following thrombectomy (Boers et al., 2019; Okamoto et al., 2021). Larger studies are required to confirm the findings of our study. Clot composition and etiology have impact on rates of recanalization, repeated occlusion, and mortality (Sun et al., 2019; Fitzgerald et al., 2021; Kim et al., 2021; Ko, 2021; Deniz et al., 2022). It is important to note that we did not do a sub-analysis of type of occluding thrombus and thrombus composition in our study. While atherothrombotic strokes have greater collateral flow and better outcomes, especially in longer onset to treatment time, this finding has not been consistent across studies (Rebello et al., 2017; Casetta et al., 2025; Yin et al., 2025). Additionally, carotid terminus occlusions could negatively influence outcomes (Linfante et al., 2002). Similar to prior reports, IC+ infarction was associated with hemorrhagic transformation of putamen, higher NIHSS score at 7 days or discharge, and worse functional outcome at discharge (Kaesmacher et al., 2021). Patients with IC+ infarction are likely to have hemorrhagic transformation of the putamen from severe damage following prolonged ischemia breaching the resistance capacity of white matter tissue (Kaesmacher et al., 2021). Although we did not do a deeper analysis of individual components of the NIHSS score, we hypothesize that a high NIHSS score in those with IC+ infarction was from the motor deficits.

Timely mechanical thrombectomy is the standard of care for proximal MCA occlusion. The goal of the study was to help clinicians identify factors associated with IC+ infarction despite endovascular thrombectomy and its impact on outcomes that is specific to our patient population. The strengths of this study include the large sample size. The limitations of our study include the single-center study, retrospective review of medical records, and the lack of consistent follow-up assessments, which could introduce bias. We lacked information on the presence of tandem occlusions, which are associated with worse outcomes from stroke recurrence, infarct progression, and challenges in management which includes no stenting, angioplasty, and stenting. Additionally, we did not explore various thrombectomy techniques, devices, especially the use of stent retrievers or suction thrombectomy, number of passes, peri-procedural management such as the use of conscious sedation as opposed to intubation with mechanical ventilation, and blood pressure targets that have a huge impact on functional outcome.

## Conclusion

The state of perfusion status of LSA prior to thrombectomy, baseline ASPECTS, presence of collateral circulation, and successful reperfusion played a crucial role in outcome following thrombectomy. Despite the development of IC+ infarction and hemorrhagic transformation of lenticulostriate tissue, delayed neurological improvement is possible. Large prospective studies are required to confirm these findings.

## Data Availability

The datasets presented in this article are not readily available because IRB approval was not obtained for data sharing of deidentified data. Requests to access the datasets should be directed to archanahinduja@yahoo.com.
